# Belladonna in Orchitis

**Published:** 1871-11

**Authors:** 


Belladonna in Orchitis.—S. D. Turney sends the following to the American Practitioner: "I often meet in the medical journals with various methods of treating orchitis. All that I have seen necessitate much time and confinement to bed, and some advise a painful surgical operation. I beg now to recommend a treatment which an experience in its use of over sixteen years warrants me in saying is both safe, speedy, and sure. It consists in the local application of the following: Extract of belladonna, one drachm; glycerole of starch, one ounce. Mix and rub well over the entire scrotum and along the cord on the affected side every three or four hours. In a short time the pain and distress abate, usually at the end of twenty-four hours. The patient, after applying a well-fitting suspensory bandage, may leave his room. The same remedy to the mamma and around the nipple will almost invariably prevent mammary abscess, if used at an early period of the inflammation. * * * The belladonna is supposed to act by constringing the minute blood-vessels of the congested parts; but whether this is so or not, the fact is unquestionable, that when used early, it will cut short inflammation in the organs referred to, and when appied at a later period, it will usually effect a speedy cure."

